# A transcriptomic approach to study the effect of long-term starvation and diet composition on the expression of mitochondrial oxidative phosphorylation genes in gilthead sea bream (*Sparus aurata*)

**DOI:** 10.1186/s12864-017-4148-x

**Published:** 2017-10-11

**Authors:** Jonás I. Silva-Marrero, Alberto Sáez, Albert Caballero-Solares, Ivan Viegas, María Pilar Almajano, Felipe Fernández, Isabel V. Baanante, Isidoro Metón

**Affiliations:** 1 Secció de Bioquímica i Biologia Molecular, Departament de Bioquímica i Fisiologia, Facultat de Farmàcia i Ciències de l’Alimentació, Universitat de Barcelona, Joan XXIII 27-31, 08028 Barcelona, Spain; 2 Departament d’Ecologia, Facultat de Biologia, Universitat de Barcelona, Diagonal 645, 08028 Barcelona, Spain; 3 Center for Neuroscience and Cell Biology (CNC), University of Coimbra, Largo Marquês de Pombal, 3004-517 Coimbra, Portugal; 4 Center for Functional Ecology (CFE), Department Life Sciences, University of Coimbra, Calçada Martins de Freitas, 3000-456 Coimbra, Portugal; 5 Departament d’Enginyeria Química, Universitat Politècnica de Catalunya, Diagonal 647, 08028 Barcelona, Spain

**Keywords:** Transcriptome, Microarray, Starvation, Diet composition, Oxidative phosphorylation, *Sparus aurata*

## Abstract

**Background:**

The impact of nutritional status and diet composition on mitochondrial oxidative phosphorylation (OXPHOS) in fish remains largely unknown. To identify biomarkers of interest in nutritional studies, herein we obtained a deep-coverage transcriptome by 454 pyrosequencing of liver and skeletal muscle cDNA normalised libraries from long-term starved gilthead sea bream (*Sparus aurata*) and fish fed different diets.

**Results:**

After clean-up of high-throughput deep sequencing reads, 699,991 and 555,031 high-quality reads allowed de novo assembly of liver and skeletal muscle sequences, respectively (average length: 374 and 441 bp; total megabases: 262 and 245 Mbp). An additional incremental assembly was completed by integrating data from both tissues (hybrid assembly). Assembly of hybrid, liver and skeletal muscle transcriptomes yielded, respectively, 19,530, 11,545 and 10,599 isotigs (average length: 1330, 1208 and 1390 bp, respectively) that were grouped into 15,954, 10,033 and 9189 isogroups. Following annotation, hybrid transcriptomic data were used to construct an oligonucleotide microarray to analyse nutritional regulation of the expression of 129 genes involved in OXPHOS in *S. aurata*. Starvation upregulated cytochrome c oxidase components and other key OXPHOS genes in the liver, which exhibited higher sensitive to food deprivation than the skeletal muscle. However, diet composition affected OXPHOS in the skeletal muscle to a greater extent than in the liver: most of genes upregulated under starvation presented higher expression among fish fed a high carbohydrate/low protein diet.

**Conclusions:**

Our findings indicate that the expression of coenzyme Q-binding protein (COQ10), cytochrome c oxidase subunit 6A2 (COX6A2) and ADP/ATP translocase 3 (SLC25A6) in the liver, and cytochrome c oxidase subunit 5B isoform 1 (COX5B1) in the liver and the skeletal muscle, are sensitive markers of the nutritional condition that may be relevant to assess the effect of changes in the feeding regime and diet composition on fish farming.

**Electronic supplementary material:**

The online version of this article (10.1186/s12864-017-4148-x) contains supplementary material, which is available to authorized users.

## Background

A major advantage of next-generation sequencing (NGS) technology is the ability to provide a huge amount of gene expression data due to its high throughput [1–3]. Among NGS approaches, massive 454 sequencing has become a feasible method for increasing sequencing depth and coverage. Following the pyrosequencing method [4], sequencing error levels in the 454 platform are low (< 1%), arising primarily because of homopolymer runs, but these errors tend to be resolved with sufficient coverage depth to allow assembly of overlapping reads. Expressed sequence tag (EST) projects in fish started in the late 90s with the model species zebrafish, *Danio rerio* [5, 6] and medaka, *Oryzias latipes* [7]. More recently, transcriptomic analyses have been developed for fish species of interest in aquaculture such as Atlantic cod, *Gadus morhua* [8–10], Atlantic salmon, *Salmo salar* [11, 12], common carp, *Cyprinus carpio* [13], rainbow trout, *Oncorhynchus mykiss* [14], Senegalese sole, *Solea senegalensis*, and common sole, *Solea solea* [15], and gilthead sea bream larvae, *Sparus aurata* [16–18], among others. *S. aurata* is the most cultured marine fish in Europe, accounting for 46% of total aquaculture production of marine fish in 2015 [19]. Despite the commercial interest of *S. aurata* production, currently available transcriptomic data for *S. aurata* provide partial information and limited support to identify genes of potential biotechnological interest in nutritional studies. Remarkable efforts to obtain transcriptomic data from *S. aurata* juveniles have been carried out by Calduch-Giner et al. [20] through sequencing 454 normalised libraries of skeletal muscle, intestine, blood and head kidney (prior and post exposure to infection with the myxosporean parasite *Enteromyxum leei*) RNA samples of *S. aurata* fed with commercial diets, combined with previous data obtained from animals exposed to confinement stress, parasite infection and nutritional stress by essential fatty acid deficiency. The authors yielded 125,263 unique sequences with an average length of 727 bp encoding for 21,384 gene descriptions. Assembled contigs ranged from 7808 to 14,008 depending on the tissue library. Additional contributions were performed by Garcia de la Serrana et al. [21], who analysed the skeletal muscle transcriptome of fish fed a commercial diet and submitted to short-term fasting at various rearing temperatures; Vieira et al. [22], who analysed the skeleton transcriptome by sequencing RNA samples from vertebrae and gill arches; and Sarropoulou et al. [18], who sequenced brain samples of *S. aurata* submitted to early-life events during the larval phase. To our knowledge, no tissues from *S. aurata* kept under different nutritional conditions such as long-term starvation and partial substitution of dietary protein by carbohydrates and lipids have been included for the generation of cDNA libraries and sequencing of ESTs collections using NGS. Therefore, current publicly available data for *S. aurata* is most likely to underrepresent genes involved in metabolic adaptation to long-term starvation and changes in the composition of dietary nutrients.

Oxidative phosphorylation (OXPHOS) is essential for transferring energy from substrate oxidation to ATP production in mitochondria, providing approximately 90% of the cellular energy. The OXPHOS system comprises complexes I to IV of the respiratory electron transport chain, which generates an electrochemical proton gradient by pumping protons across the inner mitochondrial membrane with the aid of ubiquinone (CoQ) and cytochrome c as mobile electron carriers, and the reversible proton pump F1F0-ATP synthase (complex V), which couples proton reflux into the mitochondrial matrix to generate ATP from ADP and phosphate. Cytochrome c oxidase (or complex IV) catalyses the terminal step of the electron transport chain (reduction of molecular oxygen to water), which is thought to be the rate-limiting reaction of the pathway [23, 24]. Starvation increases the rate of oxygen consumption at about 20% in the rat liver [25], and enhances cytochrome c oxidase activity in the liver of mice [26]. Starvation increases OXPHOS activity in the liver of mice by stimulating the transcription and efficiency of OXPHOS genes in a process triggered by glucagon/cAMP signalling [27]. Similarly, starvation impairs the glycolytic flux, reduces the ATP/AMP ratio and significantly enhances the activity of cytochrome c oxidase in human fibroblasts [28]. Indeed, the ATP/ADP ratio is considered a major regulator of the phosphorylation status and activity of cytochrome c oxidase [24]. In fish, knowledge of the effect of starvation on the OXPHOS pathway remains limited. In a recent report, Bermejo-Nogales et al. [29] reported the effect of 10 days of starvation on the expression of 88 genes of the OXPHOS pathway in the liver, white skeletal muscle and cardiac muscle of *S. aurata* juveniles. In contrast to previous observations in mammals, 10 days of starvation downregulated most of OXPHOS genes in the liver of food-deprived fish, while upregulated the expression of some OXPHOS genes in the white skeletal muscle and cardiac muscle.

Little is currently known about the effect of macronutrient composition of the diet on the expression of OXPHOS genes. Accumulating evidence indicates that high-fat diets decrease the expression, activity and assembly of the five OXPHOS complexes in the liver and skeletal muscle of mice [30–32], and partial substitution of dietary protein and fat by starch significantly increases mRNA levels of OXPHOS markers such as ubiquinol-cytochrome c reductase subunit 2 (UCR2) and cytochrome c oxidase subunit 4 (COX4) in the liver of rainbow trout (*Oncorhynchus mykiss*), although with expression changes <2-fold in both cases [33].

The aim of the present study was to perform massive 454 sequencing of liver and white skeletal muscle cDNA libraries from *S. aurata* juveniles submitted to different nutritional conditions (long-term starvation and feeding diets differing in nutrient composition) in order to provide a deep-coverage RNA sequencing data for developing nutritional studies in fish. The resulting database was used to design an oligonucleotide microarray and analyse the effect of nutritional status and diet composition on the expression of genes involved in OXPHOS in *S. aurata*.

## Methods

### Animals, feeding trial, sampling and growth performance

Gilthead sea bream (*S. aurata*) juveniles were obtained from Piscimar (Burriana, Castellón, Spain), transported to the laboratory, and distributed into 260 L aquaria maintained at 21 °C and supplied with running seawater in a closed system with active pump filter and UV lamps. The photoperiod was adjusted to a 12 h: 12 h dark-light cycle. Acclimation to our facilities and maintenance procedures were as previously described [34]. Previous to sampling and to maximise representation and diversity of transcripts of nutritional interest in the cDNA libraries used for 454-pyrosequencing, fish were submitted to six different nutritional conditions. Five groups of fish were fed at a ration of 25 g/kg body weight once a day (10 a.m.) for 23 days with diets that were formulated to cover a range of macronutrients above and below the levels in commercially available diets. Nutrient composition was adjusted to achieve similar energy levels (20 to 22 kJ/g). Diets were named HLL, MHL, MLH, LHH and LLH, where the first, second and third letters indicate the levels of protein, lipids and carbohydrates, respectively (H: high; M: medium; L: low). The composition of the experimental diets is shown in Table 1. A sixth group of fish was deprived of food during the same period of time. To prevent stress during tissue sampling, fish were anesthetised with MS-222 (1:12,500) before handling (9:30 a.m.) and killed by cervical section. Blood was collected and tissue samples (liver and white skeletal muscle extracted from the middle/dorsal region) were dissected out, immediately frozen in liquid N_2_, and kept at −80 °C until use. Specific growth rate (SGR, %/day) was calculated according to the following expression: SGR = (ln Wf − ln Wi) * 100/T, where Wf and Wi are final and initial weight in grams, respectively, and T is time in days.

**Table 1:** Composition of the diets supplied in this study to *S. aurata*

	HLL	MHL	MLH	LHH	LLH
Formulation (%)					
Fish meal^a^	81.6	67.6	67.6	54.3	54.3
Fish oil^b^	0.8	13.1	3.1	16.5	6.0
Starch^c^	15.0	16.7	26.7	26.6	37.1
Vitamin mixture^d^	0.2	0.2	0.2	0.2	0.2
Mineral mixture^e^	0.9	0.9	0.9	0.9	0.9
Carrageenan^f^	1.5	1.5	1.5	1.5	1.5
Proximate composition (%)					
Protein	58.0	48.0	48.0	38.6	38.6
Carbohydrates^g^	15.0	16.7	26.7	26.6	37.1
Fat	9.9	20.7	10.7	22.5	12.1
Ash	15.4	12.9	12.9	10.5	10.5
Gross energy (kJ/g)^h^	20.1	22.0	20.0	22.1	20.0

^a^Corpesca S.A. Super-Prime fish meal (Santiago de Chile, Chile)

^b^Fish oil from A.F.A.M.S.A. (Vigo, Spain)

^c^Pregelatinised corn starch from Brenntag Química S.A. (St. Andreu de la Barca, Barcelona, Spain)

^d^Vitamin mixture provided (mg/Kg): choline chloride, 1200; myo-inositol, 400; ascorbic acid, 200; nicotinic acid, 70; all-rac-tocopherol acetate, 60; calcium pantothenate, 30; riboflavin, 15; piridoxin, 10; folic acid, 10; menadione, 10; thiamin-HCl, 8; all-trans retinol, 2; biotin, 0,7 cholecalciferol, 0.05; cyanocobalamin, 0.05

^e^Mineral mixture provided (mg/Kg): CaHPO_4_.2H_2_O, 7340; MgO, 800; KCl, 750; FeSO_4_.7H_2_O, 60; ZnO, 30; MnO_2_, 15; CuSO_4_.5H_2_O, 1.7; CoCl_2_.6H_2_O, 1.5; KI, 1.5; Na_2_SeO_3_, 0.3

^f^Iota carrageenan (Sigma-Aldrich)

^g^Carbohydrates were calculated by difference

^h^Calculated from gross composition (protein 24 kJ/g, lipids 39 kJ/g, carbohydrates 17 kJ/g)

### RNA extraction, cDNA library construction, normalisation and 454 sequencing

Total RNA was extracted from 30 mg of liver or white skeletal muscle using the RNeasy tissue and RNeasy fibrous tissue, respectively, mini kits (Qiagen, Hilden, Germany) according to the manufacturer’s instructions. RNA concentration and purity was determined spectrophotometrically at 260/280 nm using Nanodrop ND-1000 (Thermo Fischer Scientific, Waltham, MA, USA). RNA integrity was determined with an Agilent 2100 bioanalyzer (Agilent Technologies, Santa Clara, CA, USA). Only samples with RNA Integrity Number (RIN) > 9.2 were used for subsequent studies. Pools consisting of 1 μg of total RNA isolated from 6 individuals per condition (fasting and feeding with diets HLL, MHL, MLH, LHH and LLH; 36 fish and RNA samples in total) were used to construct liver and white skeletal muscle cDNA libraries. The dsDNA synthesis was performed using a MINT-Universal cDNA synthesis kit (Evrogen, Moscow, Russia). To increase the presence of rare transcripts, the cDNAs libraries were normalised using TRIMMER cDNA normalization kit (Evrogen, Moscow, Russia) following manufacturer’s instructions. Sequencing of cDNAs libraries was performed using a GS FLX 454 platform (Roche, Basel, Switzerland) at the CCiTUB of the Universitat de Barcelona (Barcelona, Spain).

### Transcriptome assembly and annotation

Pre-processing of raw reads to remove low quality bases, primers and adapters and quality control were performed using Cutadapt [35], Prinseq [36] and FastQC (Babraham Bioinformatics, Cambridge, UK). The reads were de novo assembled using the GSAssembler software (Roche, Basel, Switzerland). Three assemblies were performed: one for sequence data from liver, another for white skeletal muscle, and an incremental assembly using both liver and white skeletal muscle data (hybrid assembly). Gene annotation of unique sequences for the three assemblies was performed with local BLASTx and BLASTn searches against non-redundant protein and nucleotide sequence databases of the NCBI’s QBLAST using GPRO software [37] with a significant threshold of E-value <1e-4. Gene names and putative functions were assigned to each sequence based on the highest alignment score among BLAST best 10 matches. GPRO software was also used to perform functional analysis by mapping annotated unique sequences to the Gene Ontology (GO) database. The number of sequences associated to GO terms was calculated under the categories of biological process, molecular function and cellular component.

### Microarray design, hybridization and data analysis

An Agilent custom high-density oligonucleotide microarray (8 × 60 k; ID 079501; Agilent Technologies, Santa Clara, CA, USA) was designed to contain 2 different 60-mer probes for each of the 25,392 assembled unique sequences present in the *S. aurata* hybrid transcriptome. Labelling, hybridisation and scanning were performed using the *Two-Color Microarray-Based Gene Expression Analysis v. 6.5* protocol (Agilent Technologies, Santa Clara, CA, USA) according to the manufacturer’s instructions. The microarray analysis was performed using total RNA isolated from liver and skeletal muscle from four fish per condition (starved fish and fish fed diets HLL, MHL and LLH). Briefly, for each sample 200 ng of total RNA was labelled with Cy3 or Cy5 using *Low Input Quick Amp Labeling Kit*. The *Two-Color* and *RNA Spike-In Kit, Two-Color* was used to monitor microarray workflow for linearity, sensitivity and accuracy. Labelled cRNA was purified with the *RNeasy Mini Kit* (Qiagen, Hilden, Germany) and quantified using a NanoDrop spectrophotometer (Thermo Scientific, Waltham, MA, USA). After assessing successful dye incorporation and sample integrity, 2.5 μg of each labelled sample was hybridised to the custom-made oligo-microarray at 65 °C for 17 h following *Gene Expression Hybridization Kit* instructions. A double loop hybridisation with dye swap experimental design was adopted with *n* = 4 fish per condition (starvation and feeding with diets HLL, MHL and LLH) and a total of 16 hybridisations (8 per tissue) [38]. Scanning was performed using an *Agilent Microarray Scanner* G2565BA. Outlier spots and spot intensity for Cy3 and Cy5 channels were extracted using *Agilent Feature Extraction* software version 10.7. Loess and Aquantile normalisation for within-and inter-array normalisation, respectively, was applied using the R-Bioconductor package [39, 40]. Data analysis was only considered for transcriptomic annotations of OXPHOS genes with E-value <1e-10 and HSP/hit >30. A linear model analysis using Limma [41] was conducted to select differentially expressed genes between conditions.

### Quantitative real-time RT-PCR

One microgram of total RNA isolated from liver or white skeletal muscle of *S. aurata* was reverse-transcribed to cDNA using Moloney murine leukemia virus RT (Life Technologies, Carlsbad, CA, USA) for 1 h at 37 °C in the presence of random hexamer primers. The cDNA product was used for subsequent quantitative real-time PCR (qPCR). The mRNA levels of *S. aurata* NADH dehydrogenase [ubiquinone] 1 beta subcomplex subunit 8 (NDUFB8), NADH ubiquinone oxidoreductase 75 kDa subunit (NDUFS1), coenzyme Q-binding protein COQ10 (COQ10), cytochrome b-c1 complex subunit 10 isoform A (UQCR11A), cytochrome c oxidase subunit 5B isoform 1 (COX5B1), cytochrome c oxidase subunit 6A2 (COX6A2), ATP synthase subunit beta (ATP5B) and ADP/ATP translocase 3 (SLC25A6), were determined in a StepOnePlus Real-Time PCR System (Applied Biosystems, Foster City, CA, USA) using 0.4 μM of each primer, 10 μl of SYBR Green (Applied Biosystems Foster City, CA, USA), and 1.6 μl of the diluted cDNA product in a final volume of 18 μl. Primers to perform qPCR analysis of the genes of interest (Table 2) were designed from sequence data obtained in the transcriptomic analysis after filtering for E-value <1e-10 and HSP/hit >30. To validate the amplification efficiency of primers, standard curves with consecutive dilutions of a cDNA test sample were generated, and PCR products were separated electrophoretically on 2% agarose gel for band size confirmation. 18S ribosomal RNA (18S) and elongation factor 1 alpha (EF1α) were selected to normalise the amount of mRNA for the genes of interest in each sample using primer pairs shown in Table 2. Variations in gene expression were calculated by the standard ΔΔCt method.

**Table 2:** Primers used to analyse gene expression by qPCR

Gene	Accession no. (GenBank)	Name of primer	Sequence (5′ to 3′)
NDUFB8	MF438220	JA1511	ATATCCCGACAAAGGCGAGGGC
NDUFB8		JA1512	AGGTCAGGGTGGTCCCACTTGT
NDUFS1	MF438168	ASA205F	ACCATTGCTCAGAACGCCAGAAC
NDUFS1		ASA205R	GGCTTTCTTTAGGCAGGTCAGCT
COQ10	MF438156	JA1505	CCAGCAAAACGACTCCACTCCTC
COQ10		JA1506	CCCACAGGAGCCCAAGTTTCT
UQCR11A	MF438251	JA1509	TATTCTGAGGGCGTGGGTGC
UQCR11A		JA1510	TCGAGAAATAAGCGCCAGTCTGT
COX5B1	MF438221	JA1513	CCTTCCTGCGGTTCCCACTA
COX5B1		JA1514	CATGAAGGAGGCAAATATGAATGC
COX6A2	MF438235	JS1703	GGGTTCGTGTGAGGGTTGTGG
COX6A2		JS1704	CATCCCTGGTGTTACTGTCTGC
ATP5B	MF438166	JA1507	GGGCAGGGTCAGTCAAATCGTCAG
ATP5B		JA1508	CAACATCTTCCGCTTCACACAGGCT
SLC25A6	MF438196	JA1503	CTGTGTTTCGTCTACCCCCTCG
SLC25A6		JA1504	CTTCACCAAACAGTCTCCCAGGC
18S	AM490061	JDRT18S	TTACGCCCATGTTGTCCTGAG
18S		DTRT18AS	AGGATTCTGCATGATGGTCACC
EF1α	AF184170	AS-EF1Fw	CCCGCCTCTGTTGCCTTCG
EF1α		AS-EF1Rv	CAGCAGTGTGGTTCCGTTAGC

### Statistics

Data obtained by performing qPCR were analysed by one-way ANOVA using the SPSS software Version 22 (IBM, Armonk, NY, USA) and are represented as mean ± standard deviation. When statistical significance was found for ANOVA, the Student-Newman-Keuls post hoc test was used to determine differences among treatments.

## Results

### 454 pyrosequencing and assembly

**Fig. 1:**
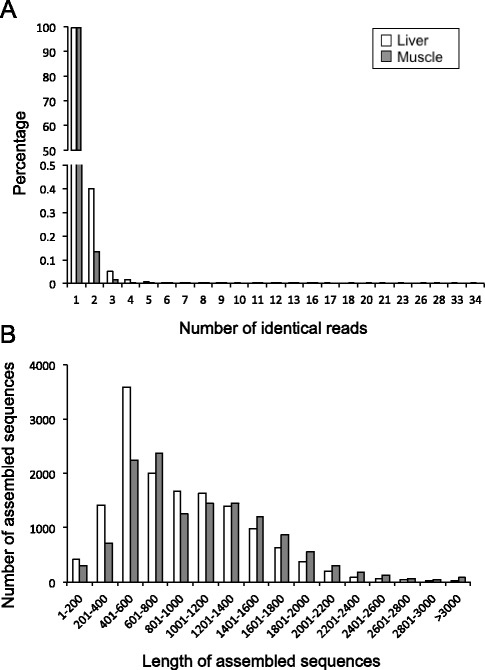
Characteristics of 454-pyrosequencing reads and unique sequences for *S. aurata* liver and skeletal muscle transcriptomes. **a** Frequency distribution of identical 454-pyrosequencing reads of liver and skeletal muscle samples. **b** Average length distribution of assembled unique sequences of liver and skeletal muscle transcriptomes

To obtain a deep-coverage database useful for developing nutritional studies in *S. aurata*, total RNA pools were made from the liver and skeletal muscle of 23-day starved fish or fish fed with five different diets for the same time period. The experimental diet HLL (High protein, Low lipids, Low carbohydrates) has a macronutrient composition similar to the diet of wild *S. aurata*. The other diets presented partial substitution of protein by lipids and/or carbohydrates. Liver and white skeletal muscle cDNA libraries were constructed, normalised to increase the presence of rare transcripts, and subsequently sequenced using the 454 FLX technology. Two sequencing runs of liver and skeletal muscle libraries yielded 812,770 and 691,433 reads, respectively. After clean-up and removal of primers and adapter sequences, sequencing of liver and white skeletal muscle libraries generated 699,991 and 555,031 high-quality reads with an average length of 374 and 441 bp and a total of 262 and 245 Mbp sequence data, respectively. Three assemblies were performed: liver, white skeletal muscle and an incremental assembly by integrating data from liver and white skeletal muscle (hybrid transcriptome). A summary of the 454 assemblies is listed in Table 3. Unique reads represented 96.8 and 96.4% of total reads of liver and skeletal muscle sequencing results, respectively (Fig. 1a). Assembly of the hybrid transcriptome was performed by aligning 1,119,088 reads (89.2% of high quality reads), which generated 23,956 contigs with average and N50 lengths of 901 bp and 1322 bp, respectively, and 102,569 singletons. Contigs were grouped into 19,530 isotigs with average and N50 lengths of 1330 and 1536 bp, respectively, and isotigs were further grouped into 15,954 isogroups. For the liver and skeletal muscle, aligned reads (88.6% and 91.2% of high quality reads, respectively) generated 13,313 and 12,333 contigs with an average length of 934 bp (N50 = 1195 bp) and 1056 bp (N50 = 1364 bp), and 43,460 and 30,302 singletons, respectively. The number of isotigs constructed was 11,545 with average and N50 lengths of 1208 bp and 1315 bp, respectively, for the liver, and 10,599 isotigs with average and N50 lengths of 1390 bp and 1491 bp, respectively, for the skeletal muscle. Isogroups in the liver and skeletal muscle transcriptomes were 10,033 and 9189, respectively (Table 3). Length distribution of assembled unique sequences is shown in Fig. 1b. Liver and white skeletal muscle transcriptomes presented 76.1 and 86.4% of unique sequences above 500 bp.

**Table 3:** Summary statistics of 454 sequencing and assembly

	Liver	Muscle	Hybrid
Data generation			
Number of reads	812,770	691,433	1,504,203
Total Megabases	298.9	275.9	574.8
Average length of reads (bp)	368	399	382
After clean-up processing			
Number of high quality reads	699,991	555,031	1,255,022
Total high quality Megabases	261.6	244.7	506.3
Average length of high quality reads (bp)	374	441	404
Assembly			
Number of contigs	13,313	12,333	23,956
Average length contigs	934	1056	901
N50 contig length	1195	1364	1322
Number of Isotigs	11,545	10,599	19,530
Average length of isotigs	1208	1390	1330
N50 isotig length	1315	1491	1536
Number of Isogroups	10,033	9189	15,954

### Annotation and gene ontology

**Fig. 2:**
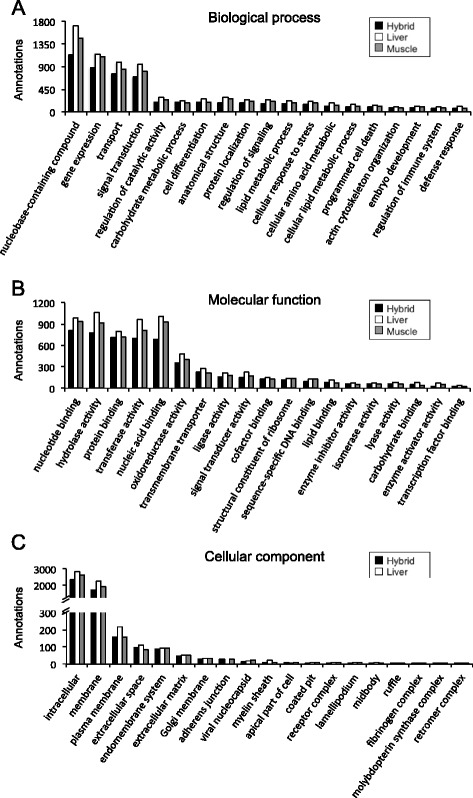
Functional gene ontology classification of *S. aurata* liver, skeletal muscle and hybrid transcriptomes. The 4th level of GO terms was used for biological process (**a**), the 3rd level for molecular function (**b**), and the 4th level for cellular component (**c**)

In the present study, 64.3%, 73.9% and 83.1% unique sequences of liver, skeletal muscle and hybrid transcriptomes, respectively, were annotated using BLASTx and BLASTn against the NCBI non-redundant protein and nucleotide collection database. To categorise gene products, 6162, 5581 and 4793 unique sequences of liver, skeletal muscle and hybrid transcriptomes, respectively, were annotated with specific GO terms. Figure 2 summarises GO terms for the assembled transcriptomes at fourth level for the categories of biological process and cellular components, and at third level for molecular function GO terms. GO terms distribution was similar for the liver, skeletal muscle and hybrid transcriptomes. Terms describing biological process were most abundant for nucleobase-containing compound metabolic process (GO:0006139; 17–19%), gene expression (GO:0010467; 13–14%), transport (GO:0006810; 11–12%), and signal transduction (GO:0007165; 10–11%) (Fig. 2a). GO terms describing molecular function were highest for nucleotide binding (GO:0000166; 12–14%), hydrolase activity (GO:0016787; 13%), protein binding (GO:0005515; 10–12%), transferase activity (GO:0016740; 12%), and nucleic acid binding (GO:0003676; 11–13%) (Fig. 2b). Most abundant GO terms describing the results for cellular components were intracellular (GO:0005622; 50–52%), membrane (GO:0016020; 37–39%), plasma membrane (GO:0005886; 3–4%), extracellular space (GO:0005615; 2%), and endomembrane system (GO:0012505; 2%) (Fig. 2c).

**Fig. 3:**
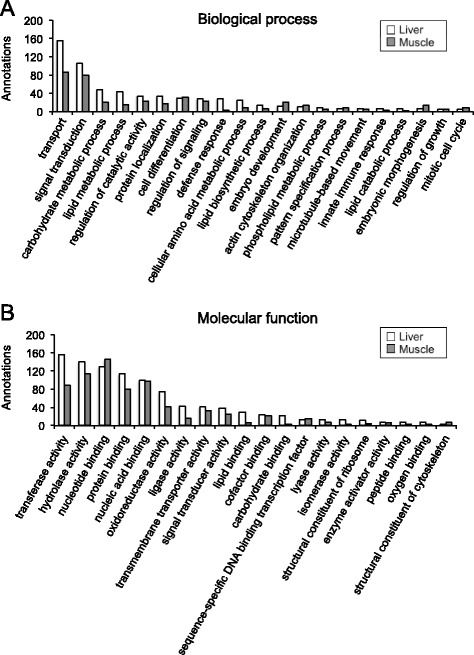
Functional gene ontology classification of annotations exclusively present in the liver and the skeletal muscle. The 4th of GO terms was used for biological process (**a**), and the 3rd level for molecular function (**b**)

GO analysis was also conducted on annotated sequences that were exclusively present either in the liver transcriptome (2457 sequences out of 19,494) or in the skeletal muscle transcriptome (2174 sequences out of 17,935). GO terms in the categories of biological process (level 4) and molecular function (level 3) are shown in Fig. 3. The more remarkable biological process GO terms showing different percentage of annotated sequences in liver versus skeletal muscle were transport (GO:0006810; 23.6 vs. 20.7%), carbohydrate metabolic process (GO:0005975; 7.1 vs. 5.0%), lipid metabolic process (GO:0006629; 6.5 vs. 3.8%), defense response (GO:0006952; 4.1 vs. 0.7%), cellular amino acid metabolic process (GO:0006520; 3.6 vs. 2.1%), cell differentiation (GO:0030154; 4.5 vs. 7.6%), embryo development (GO:0009790; 1.7 vs. 4.8%), actin cytoskeleton organisation (GO:0030036; 1.5 vs. 3.1%), pattern specification process (GO:0007389; 1.1 vs. 2.1%), embryonic morphogenesis (GO:0048598; 0.9 vs. 3.1%), and mitotic cell cycle (GO:0000278; 0.6 vs. 1.9%). With regard to molecular function, GO terms with different percentage of annotated sequences in liver and skeletal muscle included transferase activity (GO:0016740; 15.8 vs. 12.3%), oxidoreductase activity (GO:0016491; 7.5 vs. 5.7%), ligase activity (GO:0016874; 4.3 vs. 2.3%), lipid binding (GO:0008289; 2.8 vs. 0.7%), carbohydrate binding (GO:0030246; 2.1 vs. 0.3%), nucleotide binding (GO:0000166; 13.0 vs. 20.0%), and nucleic acid binding (GO:0003676; 9.9 vs. 13.4%).

**Fig. 4:**
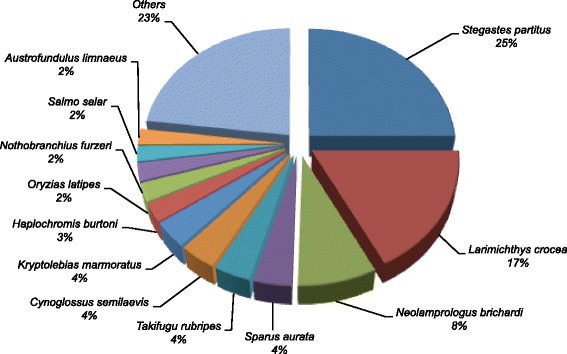
BLAST top-hit species distribution of homologous sequences to annotations in the *S. aurata* hybrid transcriptome

A BLAST top-hit species distribution of gene annotations in the hybrid *S. aurata* transcriptome showed the highest homology with NCBI non-redundant database sequences from *Stegastes partitus* (23.5%), followed by *Larimichthys crocea* (16.3%), *Neolamprologus brichardi* (7.5%), *Sparus aurata* (3.6%), *Takifugu rubripes* (3.5%), *Cynoglossus semilaevis* (3.5%) and *Kryptolebias marmoratus* (3.4%) (Fig. 4).

### Microarray and gene expression analysis

With the aim of identifying new biomarkers related to OXPHOS of interest for nutritional studies in *S. aurata*, we used the obtained transcriptomic data for designing an oligonucleotide microarray to further analyse changes in gene expression due to long-term starvation or feeding diets differing in macronutrient composition. To this end, three groups of fish were fed 23 days on diets HLL (High protein, Low lipids, Low carbohydrates; with a macronutrient composition similar to the diet of wild *S. aurata*), MHL (Medium protein, High lipids, Low carbohydrates; with a composition similar to commercial diets for *S. aurata* culture) and LLH (Low protein, Low lipids, High carbohydrates; with partial substitution of protein by carbohydrate compared to the HLL diet). A fourth group of fish was deprived of food for the same period. Growth performance of starved fish and fish fed diets HLL, MHL and LLH, expressed as mean SGR (%/day) ± SD, was −0.89^a^ ± 0.54, 2.54^c^ ± 0.21, 2.07^bc^ ± 0.33, and 1.43^b^ ± 0.51, respectively (different superscript letters indicate significant differences, *p* < 0.05; *n* = 3 groups of 8 fish each). The expression of a total of 129 genes involved in mitochondrial respiratory chain, oxidative phosphorylation and ATP translocation across the mitochondrial inner membrane were analysed in the liver and skeletal muscle of *S. aurata* (Additional file 1). Despite recent efforts to increase transcriptomic data concerning genes involved in the OXPHOS pathway in *S. aurata* [29], 38 out of 129 genes analysed in the present study are newly described genes for *S. aurata* that have not been previously reported in public databases. Long-term starvation deeply affected the expression of an important part of analysed genes. Considering statistical significance with an adjusted *P* value <0.05, 24–38 genes (depending on the diet supplied) out of 129 were significantly upregulated in the liver of starved fish, while food restriction downregulated 10–14 genes. Differentially expressed genes in the liver included 8 upregulated and 5 downregulated subunits out of 42 genes associated to NADH:ubiquinone oxidoreductase (complex I of the electron transfer chain), 1 upregulated and 2 downregulated subunits out of 7 from succinate dehydrogenase (complex II), 4 upregulated subunits out of 14 from ubiquinol-cytochrome c reductase (complex III), 15 upregulated and 4 downregulated subunits out of 34 from cytochrome c oxidase (complex IV), 5 upregulated and 2 downregulated subunits out of 19 from F1F0-ATP synthase (OXPHOS complex V), and 2 upregulated genes out of 3 ADP/ATP translocases (Additional file 2). Food deprivation resulted in 17–40 upregulated and 21–28 downregulated OXPHOS genes in the skeletal muscle (Additional file 3).

**Fig. 5:**
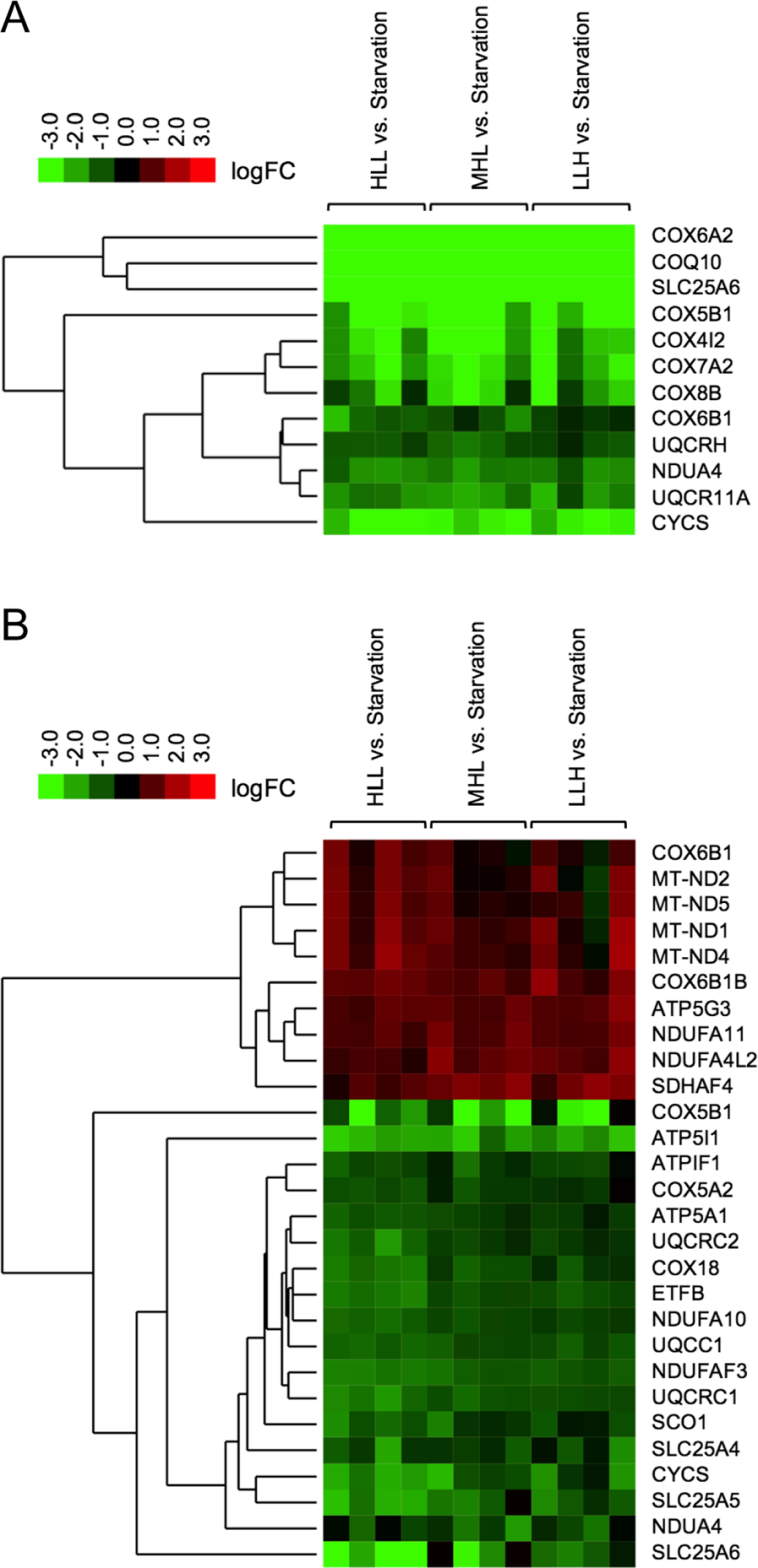
Heat map image of differentially transcribed genes involved in OXPHOS. Three groups of fish were fed 23 days at a daily ration of 25 g/kg body weight with diets HLL, MHL or LLH. A fourth group of animals was fasted for the same period. Hierarchical clustering of differentially expressed genes in the liver **a** and the skeletal muscle **b** is represented from microarray data obtained from tissue samples of *S. aurata* fed with diets HLL, MHL or LLH versus starved fish) with an adjusted *P* value <0.05 and a difference of at least 2-fold in the normalised intensity ratio (Cy5/Cy3 or Cy3/Cy5) for one or more dietary conditions. Results are presented as logFC mean value (n = 4 fish); logFC is the log2 transformed fold change. Green colour denotes downregulated genes and red colour upregulated genes in fed animals. ATP synthase subunit alpha (ATP5A1), ATP synthase F(0) complex subunit C3 (ATP5G3), ATP synthase subunit e isoform 1 (ATP5I1), ATPase inhibitor (ATPIF1), coenzyme Q-binding protein COQ10 (COQ10), cytochrome c oxidase subunit 4 isoform 2 (COX4I2), cytochrome c oxidase subunit 5A isoform 2 (COX5A2), cytochrome c oxidase subunit 6A2 (COX6A2), cytochrome c oxidase subunit 7A2 (COX7A2), cytochrome c oxidase subunit 5B isoform 1 (COX5B1), cytochrome c oxidase subunit 6B1 (COX6B1), cytochrome c oxidase subunit 6B isoform 1B (COX6B1B), cytochrome c oxidase subunit 8B (COX8B), mitochondrial inner membrane protein COX18 (COX18), cytochrome c (CYCS), electron transfer flavoprotein subunit beta (ETFB), NADH-ubiquinone oxidoreductase chain 1 (MT-ND1), NADH-ubiquinone oxidoreductase chain 2 (MT-ND2), NADH-ubiquinone oxidoreductase chain 4 (MT-ND4), NADH-ubiquinone oxidoreductase chain 5 (MT-ND5), NADH dehydrogenase [ubiquinone] 1 alpha subcomplex subunit 4 (NDUFA4), NADH dehydrogenase [ubiquinone] 1 alpha subcomplex subunit 4-like 2 (NDUFA4L2), NADH dehydrogenase [ubiquinone] 1 alpha subcomplex subunit 10 (NDUFA10), NADH dehydrogenase [ubiquinone] 1 alpha subcomplex subunit 11 (NDUFA11), NADH dehydrogenase [ubiquinone] 1 alpha subcomplex assembly factor 3 (NDUFAF3), protein SCO1 homolog (SCO1), succinate dehydrogenase assembly factor 4 (SDHAF4), ADP/ATP translocase 1 (SLC25A4), ADP/ATP translocase 2 (SLC25A5), ADP/ATP translocase 3 (SLC25A6), ubiquinol-cytochrome-c reductase complex assembly factor 1 (UQCC1), cytochrome b-c1 complex subunit 10 isoform A (UQCR11A), cytochrome b-c1 complex subunit 1 (UQCRC1), cytochrome b-c1 complex subunit 2 (UQCRC2), cytochrome b-c1 complex subunit 6 (UQCRH)

Figure 5 shows a heat map hierarchical clustering of differentially expressed genes (fish fed with diets HLL, MHL or LLH versus starved fish) with an adjusted *P* value <0.05 and with a difference of at least 2-fold in the normalised intensity ratio (Cy5/Cy3 or Cy3/Cy5) for one or more feeding conditions. With a fold change greater than 2, all differentially expressed genes were upregulated by starvation in the liver of *S. aurata*, and 6 genes corresponded to cytochrome c oxidase subunits (COX6A2, COX5B1, COX4I2, COX7A2, COX8B and COX6B1). Indeed, 4 cytochrome c oxidase components (COX6A2, COX5B1, COX4I2 and COX7A2) showed a fold change greater than 4 regardless of the diet supplied (fold change ranging from 4.9 to 86.7). In addition to cytochrome c oxidase subunits, SLC25A6 and COQ10 were also found among upregulated genes with greater fold change expression in the liver of starved fish (SLC25A6: 44.9 to 60.1 fold change depending on the diet supplied; and COQ10: 14.4 to 19.6 fold change). In contrast, starvation resulted in both upregulated and downregulated genes, 18 and 10 respectively, with a fold change greater than 2 in the skeletal muscle. The magnitude of expressional changes due to starvation was lower in the skeletal muscle than in the liver. No genes in the skeletal muscle showed a fold change greater than 4 when compared starved fish versus fish fed diets HLL, MHL and LLH. Remarkably, diet composition affected the expression of OXPHOS genes in the skeletal muscle to a greater extent than in the liver. As a general trend, fish fed LLH exhibited higher mRNA levels than fish fed HLL for the majority of significantly upregulated genes in the skeletal muscle of starved fish (Additional file 3).

**Fig. 6:**
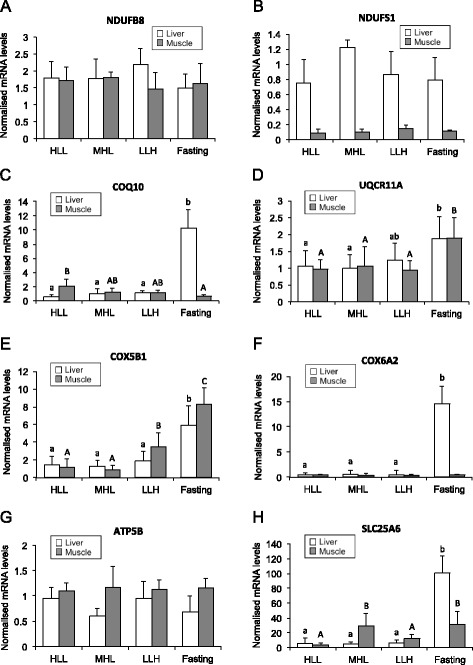
Effect of starvation and diet composition on the expression of OXPHOS genes. Three groups of fish were fed 23 days at a daily ration of 25 g/kg body weight with diets HLL, MHL or LLH. A fourth group of animals was fasted for the same period. Expression levels in the liver and the skeletal muscle are shown for NDUFB8 (**a**), NDUFS1 (**b**), COQ10 (**c**), UQCR11A (**d**), COX5B1 (**e**), COX6A2 (**f**), ATP5B (**g**) and SLC25A6 (**h**). Expression levels for each gene were normalised using 18S and EF1α as housekeeping genes. Results are presented as mean ± SD (*n* = 5 fish). Different letters (lowercase for the liver; uppercase for the skeletal muscle) indicate significant differences among conditions (*p* < 0.05)

To validate microarray data, the mRNA levels of several genes involved in OXPHOS were determined by RT-qPCR in liver and skeletal muscle samples from *S. aurata* submitted to long-term starvation or fed diets differing in macronutrient composition. The mRNA abundance of eigth genes involved in mitochondrial electron transfer chain (NDUFB8, NDUFS1, COQ10, UQCR11A, COX5B1 and COX6A2), ATP synthesis (ATP5B) and ADP/ATP translocation (SLC25A6) were analysed. The mRNA levels of NDUFB8 and NDUFS1, components of NADH:ubiquinone oxidoreductase, were not affected by the nutritional condition in the liver and the skeletal muscle. (Fig. 6a and b). The expression of COQ10, a protein required for coenzyme Q activity in the electron transport chain [42], was also determined in tissue samples of treated fish. In the liver, COQ10 mRNA significantly increased after starvation, reaching values 17-fold higher than fish fed diet HLL, and 9.3 and 10.2-fold higher than fish fed diets LLH and MHL, respectively. A different regulation of COQ10 expression was observed in the skeletal muscle by the nutritional condition: a significant 3-fold decrease was found in starved fish compared to those fed a high protein/low carbohydrate diet (HLL) (Fig. 6c).

The mRNA abundance of UQCR11A and COX5B1, components of ubiquinol-cytochrome c reductase and cytochrome c oxidase, respectively, of the respiratory chain, showed a similar behaviour as a result of starvation or feeding different diets. Starvation significantly increased the expression of both proteins in the liver (UQCR11A: 1.8 and 1.9-fold compared to fish fed HLL and MHL, respectively; and COX5B1: 3.2 to 4.5-fold compared to fed fish regardless of the diet) and the skeletal muscle (1.8 to 2.0-fold for UQCR11A and 2.4 to 9.2-fold for COX5B1 compared to fed fish regardless of the diet). Diet composition did not affect UQCR11A expression, while COX5B1 mRNA levels significantly increased 2.9 and 3.9-fold in the skeletal muscle of fish fed diet LLH compared to those fed HLL and MHL, respectively (Fig. 6d and e).

Another component of cytochrome c oxidase, COX6A2, was also greatly affected by food deprivation in the liver. Starvation significantly increased the hepatic mRNA levels of COX6A2 23.1 to 34.6-fold, depending on the diet supplied. In contrast, COX6A2 expression was not affected by the nutritional condition in the skeletal muscle (Fig. 6f). Neither starvation nor diet composition significantly affected the expression of ATP5B, a subunit of the catalytical core of F1F0-ATP synthase, in the skeletal muscle (Fig. 6g). SLC25A6, also known as ADP/ATP translocase 3, is a member of the solute carrier family 25 that facilitates the exchange of ADP and ATP across the mitochondrial inner membrane by an antiport mechanism [43]. In the liver, starvation significantly increased 14.3 to 19.3-fold the expression of SLC25A6 compared to fed fish. The effect of starvation depended on the diet composition in the skeletal muscle: fish fed diet HLL presented the lower SLC25A6 mRNA levels, which were 7.6 and 8.2-fold higher in fish fed diet MHL and starved fish, respectively (Fig. 6h).

## Discussion

### Transcriptomic analysis

The goal of the present study was to increase currently available transcriptomic data for *S. aurata* and identify OXPHOS genes affected by the nutritional status in this species. 454 sequencing of liver and white skeletal muscle cDNA normalised libraries from *S. aurata* juveniles submitted to starvation or feeding on five selected diets allowed us to generate transcriptomes for liver, skeletal muscle and a hybrid transcriptome integrating data from both tissues. The number of isotigs (19,530), contigs (23,956) and average isotig and contig length (1330 bp and 901 bp, respectively) of the hybrid transcriptome indicates that effective data assemblies were carried out compared to values reported for other fish species of interest in aquaculture [44]. In contrast to previous studies, prior to 454 pyrosequencing we constructed normalised libraries using tissue samples from long-term starved fish and fish fed five diets differing in macronutrient composition to allow maximal representation of transcripts abundance and diversity in regard to dietary condition. Furthermore, the present study reports the first liver transcriptome for *S. aurata* obtained using NGS technology. In regard of the skeletal muscle, assembly of five cDNA libraries from the fast skeletal muscle of fed and short-term fasted fish submitted to various rearing temperatures allowed Garcia de la Serrana et al. [21] to identify 50,515 isotigs with an average length of 454 bp (11,545 isotigs with an average length of 1208 bp in the present study), while Calduch-Giner et al. [20] obtained 7808 contigs with an average length of 968 bp for the same tissue (12,333 contigs with an average length of 1056 bp in the present study). The length of assembled sequences of the present study allowed annotation of a significant part of unique sequences, improving transcriptome depth.

Functional annotation allowed us to analyse frequency of GO terms in assembled transcriptomes and within annotated sequences exclusively expressed in the liver or the skeletal muscle. Concerning GO terms, liver, skeletal muscle and hybrid transcriptomes exhibited an overall similar functional profiling. However, after filtering common sequences present in the liver and skeletal muscle transcriptomes, marked differences in GO term frequency were found between both transcriptomes for biological process and molecular function categories. Consistent with the plasticity and complexity of the hepatic metabolism, the liver transcriptome presented a higher number of GO terms describing biological processes such as transport, carbohydrate metabolic process, lipid metabolic process, defense response and cellular amino acid metabolic process, and molecular functions related to enzyme activity. During growth skeletal muscle develops an adaptive response that includes tissue repair, cytoskeleton organization and remodeling processes [45]. Consistent with a higher growth rate in *S. aurata* juveniles than in adults [46], GO terms associated with biological processes involved in development and morphogenesis, such as cell differentiation, embryo development and actin cytoskeleton organisation, were more abundant in the skeletal muscle transcriptome from *S. aurata* juveniles.

BLAST similarity analysis of unique sequences in the *S. aurata* hybrid transcriptome against NCBI non-redundant database showed higher homology to genes from ten fish species belonging to the *Percomorphaceae* subdivision (68.3% of annotations), as it is the case for *S. aurata* [47]. The low number of annotations matching sequences from *S. aurata* highlights the limited genomic information available for this species in the NCBI database.

### Nutritional regulation of OXPHOS in *S. aurata*

Transcriptomic data obtained in the present study was further used to identify biomarker genes of interest for nutritional studies by analysing the effect of nutritional status and diet composition on the expression of genes involved in OXPHOS and ATP translocation across the mitochondrial inner membrane in *S. aurata*. Given that starvation periods can induce compensatory growth in commercial fish farming [48], we studied the effect of long-term starvation on the expression of OXPHOS genes in *S. aurata*. Microarray analysis and validation of microarray data by qPCR pointed to a marked increase in the mRNA levels of a high proportion of OXPHOS genes as a result of long-term starvation in the liver of *S. aurata*. In the skeletal muscle, starvation upregulated the majority of OXPHOS genes displaying changes in expression >2-fold change, although at a considerably smaller magnitude than in the liver. In contrast to the liver, food deprivation downregulated also a significant amount of OXPHOS genes in the skeletal muscle of *S. aurata*. Thus, our findings indicate that the OXPHOS process in the liver is more sensitive to long-term starvation. In support of this hypothesis, mitochondrial-related changes in fasted mice are also greater in the liver than in the skeletal muscle [49]. Indeed, it was reported that fasting does not affect the expression of OXPHOS genes in the skeletal muscle of humans [50].

Bearing in mind that cytochrome c oxidase is considered to be the rate-limiting reaction of the electron transfer chain [23, 24], the fact that starvation markedly increased the expression of multiple components of cytochrome c oxidase is consistent with upregulation of OXPHOS in the liver of food-deprived *S. aurata* (15 out of 35 genes significantly upregulated with an adjusted *P* value <0.05; 6 of them with a fold change >2). Our findings are in agreement with previous evidences highlighting increased OXPHOS activity as an important physiological adaptation in the liver during starvation. Similarly as in *S. aurata*, starvation increases cytochrome c oxidase activity and the transcription of OXPHOS genes in the liver of mice [26, 27], and enhances the activity of cytochrome c oxidase in human fibroblasts [28]. Moreover, a microarray analysis conducted on liver samples of mice indicated that 75% of diet restriction results in a modest but significant increased expression of an important number of OXPHOS genes [51]. In contrast to our findings, a recent report addressing the effect of 10 days of starvation on the expression of 88 genes of the OXPHOS pathway in *S. aurata* juveniles concluded that food-deprivation mostly downregulated the expression of OXPHOS genes in the liver, while starvation upregulated a number of OXPHOS genes in the white skeletal muscle and cardiac muscle, in most cases with a fold change <2 [29]. The starvation period (10 days versus 23 days in the present study) may explain striking differences among studies. Indeed, long periods of starvation are frequent during the life cycle of many fish species as a result of migration, reproduction and food availability [52]. Hence, metabolic adaptation to food deprivation includes a gradual depletion of hepatic glycogen levels in *S. aurata*, and starvation periods longer than 18 days are required to decrease the liver glycogen content to barely detectable levels [53, 54]. Therefore, it is not surprising that the expression of OXPHOS genes in *S. aurata* tissues could exhibit different patterns depending on the starvation period. In agreement with this hypothesis, three weeks of starvation were necessary to promote a significant increase in the protein content of succinate dehydrogenase, cytochrome c oxidase and F1F0-ATP synthase in the skeletal muscle of fine flounder (*Paralichthys adspersus*) [55]. Thus, stimulation of OXPHOS in the liver of *S. aurata* by long-term starvation may result from a metabolic adaptation that would involve increased lipolysis, enhanced hepatic fatty acid oxidation and ketogenesis, and reduced glucose uptake and oxidation in peripheral tissues. Consistently, starvation stimulates ATP-dependent processes in the liver, such as gluconeogenesis and ureagenesis, and β-oxidation of fatty acids to provide ketogenic substrates [56]. Therefore, increased OXPHOS activity may be essential to facilitate substrate oxidation and supply ATP in the liver of long-term starved *S. aurata*.

Among cytochrome c oxidase components significantly upregulated in the liver of starved *S. aurata*, COX6A2 and COX5B1 were more sensitive to food deprivation. Both components may exert an important role in cytochrome c oxidase activity during starvation. In fact, the expression of COX5B, which enhances cytochrome c oxidase activity [57], is repressed by high concentrations of glucose in *Saccharomyces cerevisiae* [58]. The hypothesis that COX6A2 expression may be relevant under food deprivation is supported by the fact that COX6A2^−/−^ mice are very sensitive to food deprivation and loose more weight during starvation than wild type mice [59]. Consistent with an increased expression of cytochrome c oxidase, the mRNA levels of cytochrome c (CYCS), a small one-electron carrier that shuttles electrons from ubiquinol-cytochrome c reductase to cytochrome c oxidase, also increased markedly in the liver of starved fish.

In addition to CYCS and cytochrome c oxidase components, upregulation of two critical proteins in mitochondrial respiration such as COQ10 and SLC25A6 closely fits with an increased OXPHOS function in the liver of starved *S. aurata*. COQ10 is a member of the steroidogenic acute regulatory protein (StAR)-related lipid transfer (START) domain superfamily that is located in the mitochondrial inner membrane, where binds ubiquinone in its hydrophobic pocket [60]. Deletion of COQ10 gene causes respiratory deficiency and the inability to oxidise NADH and succinate in yeast, while overexpression of COQ10 restores respiratory electron transport in the yeast COQ10 null mutant [42, 61–63]. These observations led to hypothesise that COQ10 is essential for mitochondrial respiration by facilitating ubiquinone biosynthesis and acting as a chaperone for the transport of ubiquinone between electron transfer chain complexes [42, 60, 63]. SLC25A6 is an ADP/ATP translocase that is ubiquitously expressed at levels that are proportional to the respiratory activity of the tissue [64]. As a core component of the mitochondrial permeability transition pore, SLC25A6 can strongly induce apoptosis in cultured human-derived cells [65, 66]. ADP/ATP translocases are integral proteins that supply cellular energy by coupling mitochondrial respiration with ADP/ATP exchange across the mitochondrial inner membrane. Presence of different ADP/ATP translocases in eukaryotic organisms may enable control of expression levels in response to a variety of stimulus and energy requirements, rather than displaying functional differences [43]. Thus, since long-term starvation caused modest effects on other ADP/ATP translocases (SLC25A4 and SLC25A5) in the liver of *S. aurata*, our findings suggest that increased mRNA levels of SLC25A6 may be critical to support ATP-dependent processes in the liver during starvation. The fact that starvation markedly upregulated COQ10, COX6A2 and SLC25A6 in the liver, while they were barely affected in the skeletal muscle, indicates that the hepatic expression of COQ10, COX6A2 and SLC25A6 can therefore be used as sensitive markers of the nutritional status in *S. aurata*.

In contrast with sensitivity of the expression of OXPHOS genes in the liver of long-term starved *S. aurata*, diet composition did not cause significant changes in the expression pattern of OXPHOS genes. Nevertheless, the expression of OXPHOS genes in the skeletal muscle was more affected by macronutrient dietary composition. Compared to fish fed HLL, the supply of a low-protein/high-carbohydrate diet (LLH) promoted a trend to upregulate mRNA levels of most of the genes whose expression significantly increased in the skeletal muscle of starved fish, such as COX5B1. Given that macronutrient composition of LLH is far from the dietary composition of wild *S. aurata* and resulted in the lowest SGR values among fed fish, our findings are consistent with the fact that partial substitution of dietary protein by carbohydrates reduces weight gain and results in expression levels of appetite-regulating peptides close to the values observed in starved *S. aurata* [67].

## Conclusions

 Four hundred fifty-four sequencing of liver and skeletal muscle samples from long-term starved *S. aurata* juveniles and fish fed diets differing in macronutrient composition allowed us to obtain a deep-coverage transcriptome. Transcriptomic data was further used to construct an oligonucleotide microarray to analyse the effect of starvation and diet composition on the expression of OXPHOS components. Our findings support the notion that long-term starvation enhances cytochrome c oxidase and OXPHOS in the liver of *S. aurata*. Among differentially expressed genes, COQ10, COX6A2 and SLC25A6 in the liver and COX5B1 in the liver and the skeletal muscle were remarkably sensitive to changes in the nutritional status, and could be useful for monitoring the impact of changes in the feeding regime and diet composition in fish.

## Additional files

Additional file 1: OXPHOS-related genes analysed in microarrays. (DOCX 151 kb)

Additional file 2: Differentially expressed genes with and adjusted *P* value <0.05 in the liver of starved *Sparus aurata* versus at least one group of fed fish (diets HLL, MHL and LLH). (DOCX 128 kb)

Additional file 3: Differentially expressed genes with and adjusted *P* value <0.05 in the skeletal muscle of starved *Sparus aurata* versus at least one group of fed fish (diets HLL, MHL and LLH). (DOCX 136 kb)

## Abbreviations

18S: 18S ribosomal RNA
ATP5A1: ATP synthase subunit alpha
ATP5B: ATP synthase subunit beta
ATP5G3: ATP synthase F(0) complex subunit C3
ATP5I1: ATP synthase subunit e isoform 1
ATPIF1: ATPase inhibitor
COQ10: Coenzyme Q-binding protein COQ10
COX18: Mitochondrial inner membrane protein COX18
COX4: Cytochrome c oxidase subunit 4
COX4I2: Cytochrome c oxidase subunit 4 isoform 2
COX5A2: Cytochrome c oxidase subunit 5A isoform 2
COX5B1: Cytochrome c oxidase subunit 5B isoform 1
COX6A2: Cytochrome c oxidase subunit 6A2
COX6B1: Cytochrome c oxidase subunit 6B1
COX6B1B: Cytochrome c oxidase subunit 6B isoform 1B
COX7A2: Cytochrome c oxidase subunit 7A2
COX8B: Cytochrome c oxidase subunit 8B
CYCS: Cytochrome c
EF1α: Elongation factor 1 alpha
EST: Expressed sequence tag
ETFB: Electron transfer flavoprotein subunit beta
GO: Gene ontology
MT-ND1: NADH-ubiquinone oxidoreductase chain 1
MT-ND2: NADH-ubiquinone oxidoreductase chain 2
MT-ND4: NADH-ubiquinone oxidoreductase chain 4
MT-ND5: NADH-ubiquinone oxidoreductase chain 5
NDUFA10: NADH dehydrogenase [ubiquinone] 1 alpha subcomplex subunit 10
NDUFA11: NADH dehydrogenase [ubiquinone] 1 alpha subcomplex subunit 11
NDUFA4: NADH dehydrogenase [ubiquinone] 1 alpha subcomplex subunit 4
NDUFA4L2: NADH dehydrogenase [ubiquinone] 1 alpha subcomplex subunit 4-like 2
NDUFAF3: NADH dehydrogenase [ubiquinone] 1 alpha subcomplex assembly factor 3
NDUFB8: NADH dehydrogenase [ubiquinone] 1 beta subcomplex subunit 8
NDUFS1: NADH ubiquinone oxidoreductase 75 kDa subunit
NGS: Next-generation sequencing
OXPHOS: Oxidative phosphorylation
qPCR: Quantitative real-time PCR
SCO1: Protein SCO1 homolog
SDHAF4: Succinate dehydrogenase assembly factor 4
SGR: Specific growth rate
SLC25A4: ADP/ATP translocase 1
SLC25A5: ADP/ATP translocase 2
SLC25A6: ADP/ATP translocase 3
START: Steroidogenic acute regulatory protein (StAR)-related lipid transfer
UCR2: Ubiquinol-cytochrome c reductase subunit 2
UQCC1: Ubiquinol-cytochrome-c reductase complex assembly factor 1
UQCR11A: Cytochrome b-c1 complex subunit 10 isoform A
UQCRC1: Cytochrome b-c1 complex subunit 1
UQCRC2: Cytochrome b-c1 complex subunit 2
UQCRH: Cytochrome b-c1 complex subunit 6
