# Supplementary material for: A transcriptomic approach to study the effect of long-term starvation and diet composition on the expression of mitochondrial oxidative phosphorylation genes in gilthead sea bream (Sparus aurata)
Source: BMC Genomics. 2017 Oct 11;18:768. doi: 10.1186/s12864-017-4148-x (PMC5637328; doi:10.1186/s12864-017-4148-x)
Supplement: Supplementary file 1 — OXPHOS-related genes analysed in microarrays. (DOCX 151 kb) [file 12864_2017_4148_MOESM1_ESM.docx]

**Additional file 1.** OXPHOS-related genes analysed in microarrays.

| Gene description | Symbol | GenBank No. |
| --- | --- | --- |
| *NADH:ubiquinone oxidoreductase complex* |  |  |
| NADH-ubiquinone oxidoreductase chain 1 | MT-ND1 | MF438141 |
| NADH-ubiquinone oxidoreductase chain 2 | MT-ND2 | MF438140 |
| NADH-ubiquinone oxidoreductase chain 4 | MT-ND4 | MF438136 |
| NADH-ubiquinone oxidoreductase chain 5 | MT-ND5 | MF438137 |
| NADH dehydrogenase [ubiquinone] 1 alpha subcomplex subunit 1 | NDUFA1 | MF438236 |
| NADH dehydrogenase [ubiquinone] 1 alpha subcomplex subunit 2 | NDUFA2 | MF438253 |
| NADH dehydrogenase [ubiquinone] 1 alpha subcomplex subunit 3 | NDUFA3 | MF438260 |
| NADH dehydrogenase 1 alpha subcomplex subunit 4 | NDUA4 | MF438229 |
| NADH dehydrogenase [ubiquinone] 1 alpha subcomplex subunit 4-like 2 | NDUFA4L2 | MF438191 |
| NADH dehydrogenase [ubiquinone] 1 alpha subcomplex subunit 6 | NDUFA6 | MF438241 |
| NADH dehydrogenase [ubiquinone] 1 alpha subcomplex subunit 7 | NDUFA7 | MF438244 |
| NADH dehydrogenase [ubiquinone] 1 alpha subcomplex subunit 8 | NDUFA8 | MF438202 |
| NADH dehydrogenase [ubiquinone] 1 alpha subcomplex subunit 9, mitochondrial | NDUFA9 | MF438190 |
| NADH dehydrogenase [ubiquinone] 1 alpha subcomplex subunit 10, mitochondrial | NDUFA10 | MF438184 |
| NADH dehydrogenase [ubiquinone] 1 alpha subcomplex subunit 11 | NDUFA11 | MF438238 |
| NADH dehydrogenase [ubiquinone] 1 alpha subcomplex subunit 12 | NDUFA12 | MF438261 |
| Acyl carrier protein, mitochondrial | NDUFAB1 | MF438161 |
| NADH dehydrogenase [ubiquinone] 1 beta subcomplex subunit 1 | NDUFB1 | MF438259 |
| NADH dehydrogenase [ubiquinone] 1 beta subcomplex subunit 2, mitochondrial | NDUFB2 | MF438237 |
| NADH dehydrogenase [ubiquinone] 1 beta subcomplex subunit 3 | NDUFB3 | MF438154 |
| NADH dehydrogenase [ubiquinone] 1 beta subcomplex subunit 4 | NDUFB4 | MF438242 |
| NADH dehydrogenase [ubiquinone] 1 beta subcomplex subunit 5, mitochondrial | NDUFB5 | MF438219 |
| NADH dehydrogenase [ubiquinone] 1 beta subcomplex subunit 6 | NDUFB6 | MF438158 |
| NADH dehydrogenase [ubiquinone] 1 beta subcomplex subunit 7 | NDUFB7 | MF438228 |
| NADH dehydrogenase [ubiquinone] 1 beta subcomplex subunit 8, mitochondrial | NDUFB8 | MF438220 |
| NADH dehydrogenase [ubiquinone] 1 beta subcomplex subunit 9 | NDUFB9 | MF438215 |
| NADH dehydrogenase [ubiquinone] 1 beta subcomplex subunit 10 | NDUFB10 | MF438218 |
| NADH dehydrogenase [ubiquinone] 1 beta subcomplex subunit 11, mitochondrial | NDUFB11 | MF438194 |
| NADH dehydrogenase [ubiquinone] 1 subunit C1, mitochondrial | NDUFC1 | MF438152 |
| NADH dehydrogenase [ubiquinone] 1 subunit C2 | NDUFC2 | MF438155 |
| NADH-ubiquinone oxidoreductase 75 kDa subunit, mitochondrial-like | NDUFS1 | MF438168 |
| NADH dehydrogenase [ubiquinone] iron-sulfur protein 2, mitochondrial | NDUFS2 | MF438172 |
| NADH dehydrogenase [ubiquinone] iron-sulfur protein 3, mitochondrial | NDUFS3 | MF438207 |
| NADH dehydrogenase [ubiquinone] iron-sulfur protein 4, mitochondrial | NDUFS4 | MF438224 |
| NADH dehydrogenase [ubiquinone] iron-sulfur protein 6, mitochondrial | NDUFS6 | MF438250 |
| NADH dehydrogenase [ubiquinone] iron-sulfur protein 7, mitochondrial | NDUFS7 | MF438193 |
| NADH dehydrogenase [ubiquinone] iron-sulfur protein 8, mitochondrial | NDUFS8 | MF438187 |
| NADH dehydrogenase [ubiquinone] flavoprotein 1, mitochondrial | NDUFV1 | MF438171 |
| NADH dehydrogenase [ubiquinone] flavoprotein 2, mitochondrial | NDUFV2 | MF438208 |
| NADH dehydrogenase [ubiquinone] flavoprotein 3, mitochondrial | NDUFV3 | MF438248 |
| Mimitin, mitochondrial | NDUFAF2 | MF438146 |
| NADH dehydrogenase [ubiquinone] 1 alpha subcomplex assembly factor 3 | NDUFAF3 | MF438205 |
| *Succinate dehydrogenase complex* |  |  |
| Succinate dehydrogenase [ubiquinone] flavoprotein subunit, mitochondrial | SDHA | MF438162 |
| Succinate dehydrogenase [ubiquinone] iron-sulfur subunit, mitochondrial | SDHB | MF438181 |
| Succinate dehydrogenase cytochrome b560 subunit, mitochondrial | SDHC | MF438216 |
| Succinate dehydrogenase [ubiquinone] cytochrome b small subunit, mitochondrial | SDHD | MF438165 |
| Succinate dehydrogenase assembly factor 1, mitochondrial | SDHAF1 | MF438157 |
| Succinate dehydrogenase assembly factor 2, mitochondrial | SDHAF2 | MF438173 |
| Succinate dehydrogenase assembly factor 4, mitochondrial | SDHAF4 | MF438209 |
| *ETF-ubiquinone oxidoreductase* |  |  |
| Electron transfer flavoprotein subunit alpha, mitochondrial | ETFA | MF438174 |
| Electron transfer flavoprotein subunit beta | ETFB | MF438188 |
| Electron transfer flavoprotein beta subunit lysine methyltransferase | ETFBKMT | MF438211 |
| Electron transfer flavoprotein-ubiquinone oxidoreductase, mitochondrial | ETFDH | MF438145 |
| *Synthesis and transport of ubiquinone* |  |  |
| Ubiquinone biosynthesis protein COQ4 homolog, mitochondrial transcript variant X1 | COQ4 | MF438195 |
| Ubiquinone biosynthesis monooxygenase COQ6, mitochondrial | COQ6 | MF438175 |
| 5-Demethoxyubiquinone hydroxylase, mitochondrial | COQ7 | MF438206 |
| Ubiquinone biosynthesis protein COQ9, mitochondrial transcript variant X2 | COQ9 | MF438151 |
| Coenzyme Q-binding protein COQ10, mitochondrial | COQ10 | MF438156 |
| *Ubiquinol-cytochrome c reductase complex* |  |  |
| Cytochrome c1, heme protein, mitochondrial | CYC1 | MF438142 |
| Cytochrome b | MT-CYB | MF438139 |
| Cytochrome b-c1 complex subunit 9 | UQCR10 | MF438239 |
| Cytochrome b-c1 complex subunit 10 isoform A | UQCR11A | MF438251 |
| Cytochrome b-c1 complex subunit 10 isoform B | UQCR11B | MF438223 |
| Cytochrome b-c1 complex subunit 7 | UQCRB | MF438240 |
| Cytochrome b-c1 complex subunit 1, mitochondrial | UQCRC1 | MF438167 |
| Cytochrome b-c1 complex subunit 2, mitochondrial | UQCRC2 | MF438176 |
| Cytochrome b-c1 complex subunit Rieske, mitochondrial | UQCRFS1 | MF438182 |
| Cytochrome b-c1 complex subunit 6, mitochondrial-like | UQCRH | MF438230 |
| Cytochrome b-c1 complex subunit 8 isoform X1 | UQCRQ | MF438257 |
| Ubiquinol-cytochrome-c reductase complex assembly factor 1 | UQCC1 | MF438170 |
| Ubiquinol-cytochrome-c reductase complex assembly factor 2 | UQCC2 | MF438245 |
| Ubiquinol-cytochrome-c reductase complex assembly factor 3 isoform X2 | UQCC3 | MF438183 |
| *Cytochrome c* |  |  |
| Cytochrome c | CYCS | MF438179 |
| Cytochrome c-type heme lyase | HCCS | MF438150 |
| *Cytochrome c oxidase complex* |  |  |
| Cytochrome c oxidase subunit 4 isoform 1, mitochondrial | COX4I1 | MF438143 |
| Cytochrome c oxidase subunit 4 isoform 2, mitochondrial | COX4I2 | MF438204 |
| Cytochrome c oxidase subunit 5A isoform 1 | COX5A1 | MF438203 |
| Cytochrome c oxidase subunit 5A isoform 2 | COX5A2 | MF438227 |
| Cytochrome c oxidase subunit 5B isoform 1 | COX5B1 | MF438221 |
| Cytochrome c oxidase subunit 6A1, mitochondrial | COX6A1 | MF438233 |
| Cytochrome c oxidase subunit 6A2, mitochondrial | COX6A2 | MF438235 |
| Cytochrome c oxidase subunit 6B1 | COX6B1 | MF438258 |
| Cytochrome c oxidase subunit 6B1 isoform A | COX6B1A | MF438226 |
| Cytochrome c oxidase subunit 6B1 isoform B | COX6B1B | MF438159 |
| Cytochrome c oxidase subunit 6C-1 | COX6C1 | MF438247 |
| Cytochrome c oxidase subunit 7A2, mitochondrial-like | COX7A2 | MF438249 |
| Cytochrome c oxidase subunit 7A2-related protein, mitochondrial | COX7A2L | MF438243 |
| Cytochrome c oxidase subunit 7B, mitochondrial | COX7B | MF438147 |
| Cytochrome c oxidase subunit 7C, mitochondrial | COX7C | MF438256 |
| Cytochrome c oxidase subunit 8A, mitochondrial | COX8A | MF438255 |
| Cytochrome c oxidase subunit 8B, mitochondrial | COX8B | MF438232 |
| Cytochrome c oxidase assembly protein COX14 | COX14 | MF438225 |
| Cytochrome c oxidase assembly protein COX15 homolog | COX15 | MF438164 |
| Cytochrome c oxidase copper chaperone | COX17 | MF438246 |
| Mitochondrial inner membrane protein COX18 | COX18 | MF438178 |
| Cytochrome c oxidase assembly protein COX19 | COX19 | MF438217 |
| Cytochrome c oxidase protein 20 homolog | COX20 | MF438197 |
| Cytochrome c oxidase subunit I | MT-CO1 | MF438138 |
| Cytochrome c oxidase assembly factor 1 homolog | COA1 | MF438263 |
| Cytochrome c oxidase assembly factor 3 homolog, mitochondrial | COA3 | MF438213 |
| Cytochrome c oxidase assembly factor 4 homolog, mitochondrial | COA4 | MF438264 |
| Cytochrome c oxidase assembly factor 5 | COA5 | MF438222 |
| Cytochrome c oxidase assembly factor 6 homolog | COA6 | MF438144 |
| Cytochrome c oxidase assembly factor 7 | COA7 | MF438177 |
| Protein SCO1 homolog, mitochondrial | SCO1 | MF438192 |
| Surfeit locus protein 1 | SURF1 | MF438199 |
| Mitochondrial import inner membrane translocase subunit Tim21 | TIMM21 | MF438185 |
| *F1F0-ATP synthase* |  |  |
| ATP synthase subunit alpha, mitochondrial | ATP5A1 | MF438153 |
| ATP synthase subunit beta, mitochondrial | ATP5B | MF438166 |
| ATP synthase subunit gamma, mitochondrial | ATP5C1 | MF438189 |
| ATP synthase subunit delta, mitochondrial | ATP5D | MF438160 |
| ATP synthase subunit epsilon, mitochondrial | ATP5E | MF438201 |
| ATP synthase F(0) complex subunit B1, mitochondrial | ATP5F1 | MF438200 |
| ATP synthase F(0) complex subunit C1, mitochondrial | ATP5G1 | MF438198 |
| ATP synthase F(0) complex subunit C3, mitochondrial | ATP5G3 | MF438214 |
| ATP synthase subunit d, mitochondrial | ATP5H | MF438234 |
| ATP synthase subunit e isoform 1, mitochondrial | ATP5I1 | MF438210 |
| ATP synthase subunit e isoform 2, mitochondrial | ATP5I2 | MF438262 |
| ATP synthase-coupling factor 6, mitochondrial | ATP5J | MF438252 |
| ATP synthase subunit f, mitochondrial | ATP5J2 | MF438148 |
| ATP synthase subunit g, mitochondrial | ATP5L | MF438254 |
| ATP synthase subunit O, mitochondrial | ATP5O | MF438212 |
| ATP synthase subunit s, mitochondrial | ATP5S | MF438149 |
| ATPase inhibitor, mitochondrial | ATPIF1 | MF438231 |
| ATP synthase mitochondrial F1 complex assembly factor 1 | ATPAF1 | MF438186 |
| ATP synthase mitochondrial F1 complex assembly factor 2 | ATPAF2 | MF438169 |
| *ADP/ATP translocases* |  |  |
| ADP/ATP translocase 1 | SLC25A4 | MF438180 |
| ADP/ATP translocase 2 | SLC25A5 | MF438163 |
| ADP/ATP translocase 3 | SLC25A6 | MF438196 |
