# Supplementary material for: A transcriptomic approach to study the effect of long-term starvation and diet composition on the expression of mitochondrial oxidative phosphorylation genes in gilthead sea bream (Sparus aurata)
Source: BMC Genomics. 2017 Oct 11;18:768. doi: 10.1186/s12864-017-4148-x (PMC5637328; doi:10.1186/s12864-017-4148-x)
Supplement: Supplementary file 2 — Differentially expressed genes with and adjusted P value <0.05 in the liver of starved Sparus aurata versus at least one group of fed fish (diets HLL, MHL and LLH). (DOCX 128 kb) [file 12864_2017_4148_MOESM2_ESM.docx]

**Additional file 2.** Differentially expressed genes with and adjusted *P* value < 0.05 in the liver of starved *Sparus aurata* *versus* at least one group of fed fish (diets HLL, MHL and LLH). Four fish per condition were used to perform microarrays. FC: fold change. NS: not significant.

| FC (HLL) | FC (MHL) | FC (LLH) | Gene symbol | Complex/function |
| --- | --- | --- | --- | --- |
| 2.99 | 2.95 | 2.71 | NDUA4 | NADH:ubiquinone oxidoreductase |
| -1.52 | -1.58 | -1.48 | NDUFA4L2 | NADH:ubiquinone oxidoreductase |
| -1.31 | -1.36 | -1.47 | NDUFA7 | NADH:ubiquinone oxidoreductase |
| NS | 1.29 | NS | NDUFA10 | NADH:ubiquinone oxidoreductase |
| -1.53 | -1.57 | -1.63 | NDUFA11 | NADH:ubiquinone oxidoreductase |
| NS | -1.33 | -1.31 | NDUFA12 | NADH:ubiquinone oxidoreductase |
| NS | 1.42 | NS | NDUFAB1 | NADH:ubiquinone oxidoreductase |
| 1.69 | 1.59 | 1.40 | NDUFB1 | NADH:ubiquinone oxidoreductase |
| 1.22 | 1.30 | NS | NDUFB5 | NADH:ubiquinone oxidoreductase |
| NS | 1.31 | NS | NDUFB8 | NADH:ubiquinone oxidoreductase |
| NS | 1.43 | 1.32 | NDUFB9 | NADH:ubiquinone oxidoreductase |
| NS | NS | -1.27 | NDUFB11 | NADH:ubiquinone oxidoreductase |
| 1.41 | NS | NS | NDUFS1 | NADH:ubiquinone oxidoreductase |
| 1.52 | 1.48 | NS | NDUFS2 | NADH:ubiquinone oxidoreductase |
| -1.35 | -1.28 | -1.55 | SDHA | Succinate dehydrogenase |
| NS | 1.32 | NS | SDHD | Succinate dehydrogenase |
| -1.53 | -1.56 | -1.76 | SDHAF2 | Succinate dehydrogenase |
| 1.35 | 1.83 | 1.61 | ETFA | ETF-ubiquinone oxidoreductase |
| 1.41 | 1.33 | 1.37 | ETFB | ETF-ubiquinone oxidoreductase |
| NS | NS | -1.69 | ETFBKMT | ETF-ubiquinone oxidoreductase |
| -1.55 | -1.49 | -1.79 | COQ9 | Synthesis and transport of ubiquinone |
| 19.64 | 18.94 | 14.36 | COQ10 | Synthesis and transport of ubiquinone |
| 2.87 | 3.33 | 2.81 | UQCR11A | Ubiquinol-cytochrome c reductase |
| NS | 1.39 | NS | UQCRC1 | Ubiquinol-cytochrome c reductase |
| NS | 1.26 | NS | UQCRFS1 | Ubiquinol-cytochrome c reductase |
| 2.00 | 2.30 | 1.75 | UQCRH | Ubiquinol-cytochrome c reductase |
| 1.28 | NS | NS | UQCC3 | Ubiquinol-cytochrome c reductase |
| 7.90 | 8.09 | 7.24 | CYCS | Cytochrome c |
| 1.49 | 1.41 | 1.27 | COX4I1 | Cytochrome c oxidase |
| 5.70 | 8.03 | 5.16 | COX4I2 | Cytochrome c oxidase |
| 1.32 | 1.38 | 1.38 | COX5A1 | Cytochrome c oxidase |
| 1.32 | 1.39 | NS | COX5A2 | Cytochrome c oxidase |
| 11.31 | 16.44 | 15.00 | COX5B1 | Cytochrome c oxidase |
| NS | 1.84 | 1.47 | COX6A1 | Cytochrome c oxidase |
| 35.01 | 86.71 | 36.25 | COX6A2 | Cytochrome c oxidase |
| 2.54 | 1.99 | NS | COX6B1 | Cytochrome c oxidase |
| 1.63 | 1.87 | 1.47 | COX6B1A | Cytochrome c oxidase |
| 1.76 | 1.82 | 1.82 | COX6B1B | Cytochrome c oxidase |
| 1.36 | 1.37 | NS | COX6C1 | Cytochrome c oxidase |
| 4.88 | 6.62 | 5.02 | COX7A2 | Cytochrome c oxidase |
| NS | 1.42 | NS | COX7B | Cytochrome c oxidase |
| NS | 4.97 | 4.36 | COX8B | Cytochrome c oxidase |
| -1.55 | -1.45 | -1.42 | COX17 | Cytochrome c oxidase |
| NS | NS | 1.46 | COX18 | Cytochrome c oxidase |
| NS | -1.75 | NS | MT-CO1 | Cytochrome c oxidase |
| -1.34 | -1.35 | -1.34 | COA5 | Cytochrome c oxidase |
| NS | 1.33 | NS | COA7 | Cytochrome c oxidase |
| NS | -1.36 | -1.32 | SURF1 | Cytochrome c oxidase |
| 1.41 | 1.52 | NS | ATP5A1 | F1F0-ATP synthase |
| 1.30 | 1.32 | NS | ATP5G1 | F1F0-ATP synthase |
| -1.23 | NS | -1.23 | ATP5G3 | F1F0-ATP synthase |
| NS | 1.24 | NS | ATP5H | F1F0-ATP synthase |
| NS | NS | 1.32 | ATP5I2 | F1F0-ATP synthase |
| -1.33 | NS | -1.32 | ATP5O | F1F0-ATP synthase |
| 1.51 | NS | NS | ATP5S | F1F0-ATP synthase |
| NS | 1.56 | 1.42 | ATPIF1 | F1F0-ATP synthase |
| 1.32 | 1.44 | 1.26 | ATPAF1 | F1F0-ATP synthase |
| 1.46 | NS | NS | SLC25A5 | ADP/ATP translocases |
| 48.90 | 60.09 | 44.87 | SLC25A6 | ADP/ATP translocases |
