# Supplementary material for: A transcriptomic approach to study the effect of long-term starvation and diet composition on the expression of mitochondrial oxidative phosphorylation genes in gilthead sea bream (Sparus aurata)
Source: BMC Genomics. 2017 Oct 11;18:768. doi: 10.1186/s12864-017-4148-x (PMC5637328; doi:10.1186/s12864-017-4148-x)
Supplement: Supplementary file 3 — Differentially expressed genes with and adjusted P value <0.05 in the skeletal muscle of starved Sparus aurata versus at least one group of fed fish (diets HLL, MHL and LLH). (DOCX 136 kb) [file 12864_2017_4148_MOESM3_ESM.docx]

**Additional file 3.** Differentially expressed genes with and adjusted *P* value < 0.05 in the skeletal muscle of starved *Sparus aurata* *versus* at least one group of fed fish (diets HLL, MHL and LLH). Four fish per condition were used to perform microarrays. FC: fold change. NS: not significant.

| FC (HLL) | FC (MHL) | FC (LLH) | Gene symbol | Complex/function |
| --- | --- | --- | --- | --- |
| -2.32 | NS | NS | MT-ND1 | NADH:ubiquinone oxidoreductase |
| -2.20 | NS | NS | MT-ND2 | NADH:ubiquinone oxidoreductase |
| -2.55 | NS | -2.02 | MT-ND4 | NADH:ubiquinone oxidoreductase |
| -2.22 | NS | NS | MT-ND5 | NADH:ubiquinone oxidoreductase |
| 1.62 | NS | NS | NDUFA1 | NADH:ubiquinone oxidoreductase |
| -1.72 | -1.23 | -1.85 | NDUFA2 | NADH:ubiquinone oxidoreductase |
| NS | -1.17 | -1.69 | NDUFA3 | NADH:ubiquinone oxidoreductase |
| NS | 2.19 | NS | NDUA4 | NADH:ubiquinone oxidoreductase |
| -1.61 | -1.26 | -2.21 | NDUFA4L2 | NADH:ubiquinone oxidoreductase |
| 1.38 | NS | NS | NDUFA8 | NADH:ubiquinone oxidoreductase |
| 1.44 | NS | NS | NDUFA9 | NADH:ubiquinone oxidoreductase |
| 2.24 | 1.81 | 1.64 | NDUFA10 | NADH:ubiquinone oxidoreductase |
| -1.81 | NS | -2.03 | NDUFA11 | NADH:ubiquinone oxidoreductase |
| NS | -1.22 | -1.47 | NDUFA12 | NADH:ubiquinone oxidoreductase |
| NS | -1.21 | -1.55 | NDUFAB1 | NADH:ubiquinone oxidoreductase |
| -1.56 | -1.14 | -1.72 | NDUFB1 | NADH:ubiquinone oxidoreductase |
| 1.56 | NS | NS | NDUFB5 | NADH:ubiquinone oxidoreductase |
| NS | NS | NS | NDUFB8 | NADH:ubiquinone oxidoreductase |
| 1.22 | NS | NS | NDUFB10 | NADH:ubiquinone oxidoreductase |
| NS | NS | -1.26 | NDUFB11 | NADH:ubiquinone oxidoreductase |
| 1.58 | NS | NS | NDUFS1 | NADH:ubiquinone oxidoreductase |
| 1.69 | NS | NS | NDUFS7 | NADH:ubiquinone oxidoreductase |
| NS | -1.13 | NS | NDUFV2 | NADH:ubiquinone oxidoreductase |
| -1.38 | 1.03 | -1.50 | NDUFV3 | NADH:ubiquinone oxidoreductase |
| 2.68 | 2.17 | 2.08 | NDUFAF3 | NADH:ubiquinone oxidoreductase |
| 1.46 | NS | NS | SDHC | Succinate dehydrogenase |
| 1.62 | NS | NS | SDHD | Succinate dehydrogenase |
| NS | -1.23 | -1.56 | SDHAF2 | Succinate dehydrogenase |
| -1.68 | 2.56 | -2.48 | SDHAF4 | Succinate dehydrogenase |
| 2.68 | 1.88 | 1.91 | ETFB | ETF-ubiquinone oxidoreductase |
| 1.33 | NS | NS | COQ4 | Synthesis and transport of ubiquinone |
| 1.81 | 1.45 | 1.43 | COQ6 | Synthesis and transport of ubiquinone |
| NS | -1.83 | -1.65 | COQ7 | Synthesis and transport of ubiquinone |
| 1.72 | NS | NS | COQ9 | Synthesis and transport of ubiquinone |
| 1.94 | 1.65 | NS | CYC1 | Ubiquinol-cytochrome c reductase |
| -1.39 | -1.15 | -1.45 | MT-CYB | Ubiquinol-cytochrome c reductase |
| 1.87 | 1.87 | NS | UQCR11A | Ubiquinol-cytochrome c reductase |
| -1.39 | -1.20 | -1.65 | UQCR11B | Ubiquinol-cytochrome c reductase |
| 2.84 | 2.09 | 1.86 | UQCRC1 | Ubiquinol-cytochrome c reductase |
| 2.65 | 1.64 | 1.57 | UQCRC2 | Ubiquinol-cytochrome c reductase |
| 1.78 | 1.61 | 1.59 | UQCRFS1 | Ubiquinol-cytochrome c reductase |
| 2.24 | 1.87 | 1.92 | UQCC1 | Ubiquinol-cytochrome c reductase |
| -1.43 | -1.11 | -1.73 | UQCC2 | Ubiquinol-cytochrome c reductase |
| 1.23 | NS | NS | UQCC3 | Ubiquinol-cytochrome c reductase |
| 3.42 | 2.40 | 2.22 | CYCS | Cytochrome c |
| 1.80 | NS | NS | HCCS | Cytochrome c |
| 1.34 | NS | NS | COX5A1 | Cytochrome c oxidase |
| 1.92 | 2.41 | NS | COX5A2 | Cytochrome c oxidase |
| NS | 8.10 | NS | COX5B1 | Cytochrome c oxidase |
| -1.73 | -1.11 | -1.69 | COX6A1 | Cytochrome c oxidase |
| -2.08 | NS | NS | COX6B1 | Cytochrome c oxidase |
| -2.02 | -1.10 | -2.08 | COX6B1B | Cytochrome c oxidase |
| NS | -1.46 | -1.60 | COX7B | Cytochrome c oxidase |
| NS | NS | -1.38 | COX7C | Cytochrome c oxidase |
| -1.70 | -1.29 | -1.80 | COX8A | Cytochrome c oxidase |
| -1.49 | -1.21 | -1.55 | COX14 | Cytochrome c oxidase |
| -1.45 | -1.26 | -1.57 | COX17 | Cytochrome c oxidase |
| 2.56 | 1.90 | 1.60 | COX18 | Cytochrome c oxidase |
| 1.45 | NS | NS | COX20 | Cytochrome c oxidase |
| -1.51 | -1.13 | -1.45 | MT-CO1 | Cytochrome c oxidase |
| 1.48 | 1.39 | NS | COA5 | Cytochrome c oxidase |
| 1.86 | NS | NS | COA6 | Cytochrome c oxidase |
| 2.33 | NS | NS | SCO1 | Cytochrome c oxidase |
| 1.48 | NS | NS | SURF1 | Cytochrome c oxidase |
| 1.76 | 2.00 | 1.36 | TIMM21 | Cytochrome c oxidase |
| 2.04 | 1.63 | 1.52 | ATP5A1 | F1F0-ATP synthase |
| -1.35 | -1.29 | -1.64 | ATP5G1 | F1F0-ATP synthase |
| -1.97 | NS | -2.25 | ATP5G3 | F1F0-ATP synthase |
| 4.04 | 3.52 | 3.52 | ATP5I1 | F1F0-ATP synthase |
| NS | -1.04 | -1.71 | ATP5I2 | F1F0-ATP synthase |
| NS | -1.38 | -1.34 | ATP5J2 | F1F0-ATP synthase |
| 1.88 | 2.85 | 1.55 | ATPIF1 | F1F0-ATP synthase |
| 1.44 | NS | 1.43 | ATPAF2 | F1F0-ATP synthase |
| 2.26 | 1.77 | 1.78 | SLC25A4 | ADP/ATP translocases |
| 3.65 | 2.02 | 2.16 | SLC25A5 | ADP/ATP translocases |
| 8.51 | NS | NS | SLC25A6 | ADP/ATP translocases |
